# Finding differentially expressed regions of arbitrary length in quantitative genomic data based on marked point process model

**DOI:** 10.1093/bioinformatics/bts371

**Published:** 2012-09-03

**Authors:** Hiroshi Hatsuda

**Affiliations:** Department of Statistics, the University of Warwick, Coventry CV4 7AL, UK

## Abstract

**Motivation:** High-throughput nucleotide sequencing technologies provide large amounts of quantitative genomic data at nucleotide resolution, which are important for the present and future biomedical researches; for example differential analysis of base-level RNA expression data will improve our understanding of transcriptome, including both coding and non-coding genes. However, most studies of these data have relied on existing genome annotations and thus are limited to the analysis of known transcripts.

**Results:** In this article, we propose a novel method based on a marked point process model to find differentially expressed genomic regions of arbitrary length without using genome annotations. The presented method conducts a statistical test for differential analysis in regions of various lengths at each nucleotide and searches the optimal configuration of the regions by using a Monte Carlo simulation. We applied the proposed method to both synthetic and real genomic data, and their results demonstrate the effectiveness of our method.

**Availability:** The program used in this study is available at https://sites.google.com/site/hiroshihatsuda/.

**Contact:** H.Hatsuda@warwick.ac.uk

## 1 INTRODUCTION

Next-generation nucleotide sequencing technologies, such as Illumina, ABI SOLiD and 454 sequencing technologies, have been revolutionizing biology and medicine by providing massive amounts of quantitative genomic data at nucleotide resolution in a relatively short time and at a relatively low cost. This revolution has been making progress on, for instance, transcriptome including RNA expression (RNA-Seq) (Nagalakshmi *et al.*, 2008) and RNA expression in transcription start sites (TSS) (5′-SAGE and CAGE) (FANTOM Consortium, 2009; Taft *et al.*, 2009, protein–DNA binding (ChIP-Seq) (Robertson *et al.*, 2007)) epigenomics including DNA methylation (Lister *et al.*, 2009) and nucleosome organization (Mavrich *et al.*, 2008). These vast amounts of quantitative genomic data have led to qualitative progress in our biomedical understanding.

Because we now have large amounts of genome-wide expression data at nucleotide resolution, there is an increasing interest in differential analysis, which is a process to compare the expressions between different biological conditions, of the quantitative genomic data; however, most previous studies of the differential analysis have depended on existing genome annotations and were conducted by each gene (Anders and Huber, 2010; Robinson and Smyth, 2007; Wang *et al.*, 2010). The differential expression analysis by smaller regions is important for a research on transcript isoforms (Pan *et al.*, 2004, 2008; Sultan *et al.*, 2008) which are significant for understanding the regulation of gene transcription. Thus, it is important to develop a reliable method for differential analysis of base-level quantitative genomic data in order to proceed with the research on the regulation of gene transcription.

Therefore, in this article, we propose a novel method to find differentially expressed regions of arbitrary length in the quantitative genomic data. The presented method searches the optimal configuration of non-overlapped genomic regions with differential expression between distinct biological conditions. For this purpose, we use a point process (Baddeley *et al.*, 2006) which is a statistical theory of point patterns and can model and analyze spatial data of points in many fields, including ecology (Law *et al.*, 2009), epidemiology (Diggle, 1993), seismology (Ogata, 1999) and computer vision (Stoica *et al.*, 2004).

**Fig. 1.**
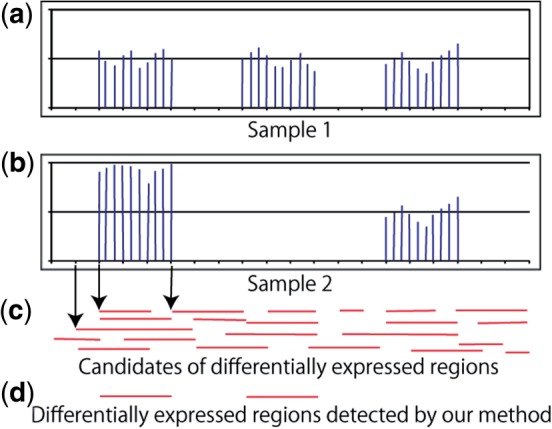
Schematic view of the proposed method. (**a**) and (**b**) Virtual expression data in a certain gene in two biological conditions. (**c**) Horizontal red line segments denote candidates of differentially expressed regions. The left edge of the segments corresponds to a nucleotide. (**d**) The proposed method finds differentially expressed non-overlapped regions from the candidates

In this study, we use one-dimensional marked point process which is an extension of a point process and has marks in each point of a point process, to model the genomic data. In our methodology, each point corresponds to each nucleotide, and the points have attributes, which are called marks, representing the genomic regions of various lengths. Figure 1 illustrates the gist of the proposed method. Figure 1a and b shows virtual expression data in a certain gene in two biological conditions. Short red line segments in Figure 1c and d denote particular regions corresponding to a specific nucleotide; there are many candidates of the regions and some of them overlap one another in Figure 1c. The proposed method removes overlapped regions and regions that are not differentially expressed; it thus finds the optimal configuration of non-overlapped regions with differential expression, as shown in Figure 1d. Note that it can detect the differentially expressed regions that are shorter in length than this gene which has almost the same expression level as a whole in the two conditions.

To search a good configuration of the regions, we define a Gibbs energy function consisting of two terms: data energy and prior energy. The data energy is based on a statistical test for differential expression analysis and the prior energy incorporates penalty for overlapping regions. The Gibbs energy is minimized by using the Reversible jump Markov chain Monte Carlo (RJMCMC) (Green, 1995) or the birth-and-death dynamics (Descombes *et al.*, 2009) coupled with the traditional simulated annealing (Metropolis *et al.*, 1953) to find the optimal configuration of the marked points. We use the birth-and-death dynamics in this study because the computational cost is much more effective in the birth-and-death dynamics than RJMCMC, and we also present a multiple birth-and-death dynamics to deal with the regions of various lengths.

Moreover, we apply a novel smoothing method (Hatsuda, 2012) based on the second-generation wavelets (Sweldens, 1997) to find candidates of the boundaries of the genomic regions prior to conducting the proposed method for finding differentially expressed regions. Although this process is not necessary in principle, it is useful to reduce the number of the candidates of the regions in advance in order to lower computational cost. The second-generation wavelet-based smoothing method is more effective than the classical wavelet-based smoothing methods because it can smooth data and yet preserve sharp edges which can be regarded as the boundaries of the regions.

In addition, the presented method is versatile because it can be used to detect any kind of regions of interest by incorporating suitable data energy and analyze every type of nucleotide-level quantitative genomic data, such as DNA methylation (Lister *et al.*, 2009) and nucleosome organization (Mavrich *et al.*, 2008) data.

## 2 RELATED WORK

Although there is no previous method for directly finding differentially expressed regions of arbitrary length, genome segmentation techniques can be used to conduct the differential analysis without genome annotations. Genome segmentation is usually performed by using *ad hoc* approaches such as a simple clustering based on sliding windows (Carninci *et al.*, 2006; Valen *et al.*, 2008); however, there are some genome segmentation techniques based on more sophisticated methods, such as hidden Markov models (HMMs) (Day *et al.*, 2007), RJMCMC (Salmenkivi *et al.*, 2002) and the graph construction of data intensity (Hower *et al.*, 2011).

Once we obtain segments that are shorter than a gene in length by genome segmentation, we can perform a statistical test for differential analysis in each segment. We thus can extract differentially expressed regions without depending on genome annotations. However, this approach has a serious limitation; genome segmentation does not divide genome based on the difference of expression between distinct biological conditions and thus involves the risk of failing to detect differentially expressed regions of arbitrary length. In contrast, the proposed method is more effective than the previous techniques because it searches them based on the difference of the expressions.

In addition, this is the first study of a practical application of marked point process to the comprehensive analysis of genomic data. There are some theoretical studies of marked point process in biomedical research (Chan and Zhang, 2007; Leung *et al.*, 2005). There is also an applied study of marked point process for modeling the occurrence of regulatory elements (Carstensen *et al.*, 2010); however, this is a limited application for the analysis of specific regions of genome. This point of view also indicates the significance of this study in biology and medicine.

## 3 METHODS

### 3.1 Point process

First, we consider a point process *X* in a bounded set *K* = [0, *X*_max_], which supports a set of genomic data whose size is *X*_max_. The set of configurations of points of *K* is defined on a probability space (*Ω, A,* **P**):
(1)
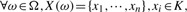

where *n* denotes the number of points related to the event *ω*. This mapping defines a point process whose random variables realize random configurations of points.

### 3.2 Marked point process

To model quantitative genomic data, we consider a marked point process. A point process is extended by adding an attribute, such as a parameter of a genomic region, to the points of a point process *X*. A marked point process *X* ′ = *X* × *M* is a point process in *X*, where each point has a mark from a bounded set *M* such as the length of the regions. Because the regions are not of the same length, we use a multiple marked point process in which the mark space *M* is associated with a set of marks, such as a set of the lengths of the regions *M* = [*γ*_min_, *γ*_max_], where *γ*_min_ and *γ*_max_ are the minimum and maximum lengths of the regions, respectively.

### 3.3 Gibbs energy

To model measurements consistent with data and interactions between marked points realized from the marked point process, we define a Gibbs density *h*(*x*) of a configuration *x* of the marked points *X* ′:
(2)
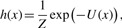

where *Z* is a normalizing constant *Z* = ∫ exp (−*U* (*x*)) *dx* and a Gibbs energy *U* (*x*) defined by
(3)
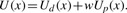

In Equation (3), the data energy *U_d_* (*x*) considers how the marked points, the regions at each nucleotide in this study, fit to the genomic data, the prior energy *U_p_*(*x*) considers the interaction of the regions, and *w* is a weighting factor between the two energy terms. The weighting factor *w* regulates the balance of the effects of the data and prior energy terms. If we emphasize the constraints on the structure of the regions based on our prior knowledge, we can limit the detection of differentially expressed regions that do not meet the conditions by setting *w* to a large value. In contrast, if we ignore the constraints and attempt to extract as many differentially expressed regions as possible based only on the data intensity, we can detect a lot of regions by setting *w* to a small value. The density *h*(*x*) will be maximized on the optimum configuration 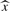. In other words, the optimum configuration can be determined by minimizing the Gibbs energy:
(4)
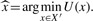

This energy minimization is feasible because the density *h*(*x*) does not need to be normalized, and the normalizing constant *Z* does not need to be computed.

### 3.4 Data energy

To define the data energy *U_d_* (*x*) to fit the marked points to the genomic data, we need to perform differential expression analysis of the regions corresponding to each nucleotide. Gene expression is often regarded as a random sampling process, and thus transcription level can be modeled by a sample from a specific probability distribution independently and uniformly in each nucleotide. Anders and Huber (2010) proposed that gene expression level in each gene can be modeled by a negative binomial distribution. The number of reads from an individual region can also be modeled by a binomial distribution and approximated by a Poisson distribution (Wang *et al.*, 2010). We use this model to conduct a statistical test for differential expression analysis in each region.

Suppose that we have *C*_1_ and *C*_2_ expressions, or read counts from a next-generation nucleotide sequencer, in a specific region for two biological conditions 1 and 2, respectively, with *C_i_* ~ **binomial**(*n_i_*,*p_i_*), *i* = 1,2, where *n_i_* denotes the total count of the reads and *p_i_* represents the probability of a read derived from that region. We then define *M* = log_2_ *C*_1_ − log_2_ *C*_2_ and 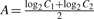, and the conditional distribution of *M* given by *A* = *a* is approximated by a normal distribution because we assume that *C*_1_ and *C*_2_ are independent based on the random sampling model. We obtain the estimates of the expectation and variance of the conditional distribution:
(5)
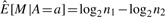

and
(6)
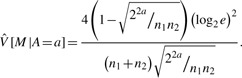

Therefore, we can calculate the *z*-score for a region *r* with *M* = *m* and *A* = *a* for a hypothesis testing *H*_0_ : *p*_1_ = *p*_2_ and *H*_1_ : *p*_1_ ≠ *p*_2_, which is given by
(7)
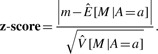

We can then convert this score to a two-sided *P*-value and determine whether the region *r* is differentially expressed or not.

We compute the data energy by converting the *z*-score to a value *U_d_* (*x_i_*) at nucleotide *i* according to
(8)
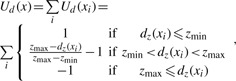

where *d_z_* (*x_i_*) = **z – score**(**region**(*x_i_*)) – *w_n_* × **z – score**(**neigbor**(*x_i_*)) and *z*_min_ and *z*_max_ are, respectively, parameters for the minimum and maximum thresholds of *z*-score. In other words, we regard the regions whose *z*-score is more than *z*_min_ as differentially expressed regions and make no distinction between the regions whose *z*-score is *z*_max_ and the regions whose *z*-score is more than *z*_max_. We compute the *z*-scores for each region and its neighboring region which is defined as adjacent *k*-mer regions on both sides, and use the weighting difference of the two *z*-scores to compute the data energy. This data energy *U_d_* (*x_i_*) provides 1 to the regions whose *z*-score is lower than *z*_min_ and negative values ∈ [–1,0] to the regions whose *z*-score is higher than *z*_min_.

### 3.5 Prior energy

To define the prior energy, we consider a penalty for overlapping and neighboring regions. We restrict neighboring regions to prevent from providing too many small regions. This prior energy is given by
(9)
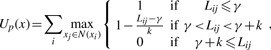

where *N* (*x_i_*) denotes a set of neighboring regions of a region, or a marked point, *x_i_*, and *L_ij_* is the distance between regions *x_i_* and *x_j_*, *γ* is the length of the region *x_i_* and *k* is a parameter for the criterion of neighborhood. We do not penalize neighboring regions whose distance is more than *k*. The computation of the prior energy takes account of all interactions between neighboring regions and selects the maximum overlap for this energy for each region. The prior energy *U_p_*(*x_i_*) ∈ [0,1] provides larger values to a region with overlapping or neighboring regions and smaller values to a region with no overlapping or neighboring regions. This energy approximates geometric constraint of the structure of the marked points in the configuration.

### 3.6 Energy minimization

To achieve the optimum configuration of the marked points, we need to minimize the Gibbs energy *U*(*x*). For this purpose, we simulate the marked point processes by using the birth-and-death dynamics, which simulates a Markov chain with only the birth transition adding a point to the configuration and the death transition removing a point from the configuration and holds the detailed balance condition in the continuous space (Descombes *et al.*, 2009).

Moreover, to deal with various lengths of the regions, we extend the birth-and-death dynamics and present a multiple birth-and-death dynamics. We define non-uniform birth rates of multiple marks based on the data energy for each marked point; we define death rates based on both the data energy and the current configuration of the marked points. The discretization scheme of the multiple birth-and-death dynamics is presented in Algorithm 1.

In addition, we use Mersenne twister (Matsumoto and Nishimura, 1998) to generate pseudo-random numbers to compare the probabilities in this algorithm.

**Algorithm 1** Multiple birth-and-death dynamics

1. Computation of the data energy: the data energy *U_d_* (*x_i_* = *m*) is computed for each nucleotide *i* ∈ *I* in the genome *I*. We compute it for each mark, and the variable *m* represents a mark *m* ∈ *M*.
2. Initialization: the inverse temperature parameter *β* and discretization step *δ* are initialized.
3. Mark selection: for each nucleotide *i* ∈ *I*, if there is no marked point, we select a mark in proportion to the rate 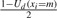.
4. Computation of the birth rate: the birth rate for each nucleotide *i* ∈ *I* is computed according to
(10)
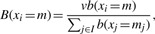

where *v* is a parameter, and *b*(*x_i_* = *m*) is given by
(11)
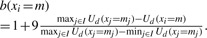

*U_d_* (*x_i_* = *m*) is the data energy at *i* whose mark is *m* selected in Step 3.
5. Birth step: for each nucleotide *i* ∈ *I* if there is no marked point and that nucleotide is included in the candidates of the start point of the regions *i* ∈ *S* described in Subsection 3.7, we add a marked point with probability *δB*(*x_i_* = *m*).
6. Computation of the prior energy: the prior energy *U_p_*(*x_i_*) of the current configuration *x* is computed for each nucleotide *i* ∈ *I*.
7. Death step: we sort the current configuration *x* from the highest to lowest data energy. In this order, the death rate *d*(*x_i_*) at each nucleotide *i* ∈ *I* is computed to
(12)
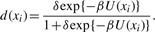

We delete a marked point at *i* with probability *d*(*x_i_*).
8. Convergence test: the convergence is achieved if the following conditions are met: all the marked points added in the birth step have been deleted in the death step, and all the marked points deleted in the death step have been added in the birth step. If the process has not converged, the temperature and the discretization step are decreased by a given factor. Then, the process goes back to Step 3.

### 3.7 Preprocessing: reducing the number of the candidates of the regions

In principle, we can successfully find differentially expressed genomic regions of arbitrary length by using the method described in Subsections 3.1–3.6. However, we perform an additional procedure to reduce the number of the candidates of the regions in order to lower the computational cost of our method. Because the starting and ending points, or edges, of the regions are probably nucleotides in which expression level substantially changes, we simply define a set of the candidates of the starting points *S* and a set of the candidates of the ending points *E* as follows:
(13)
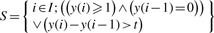

(14)
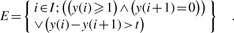

In Equations (13) and (14), *y*(*i*) represents the expression data at nucleotide *i* and *t* is a parameter for the threshold of the drastic change.

Although we can select *S* and *E* from raw data *y* based in Equations (13) and (14), we also apply a smoothing method to the data to further reduce the number of the candidates. For this purpose, we employ a novel smoothing method (Hatsuda, 2012) based on the second-generation wavelet transform (SGWT) (Sweldens, 1997), because it can smooth quantitative data but yet preserve sharp edges.

Unlike the classical wavelets, SGWT consists of the lifting scheme in which base (wavelet) functions are not based on scaling and translation of base functions but constructed based on the contents of data to cope with local particularities of the data. Therefore, SGWT realizes nonlinear data-dependent multi-scale decomposition, and the smoothing method based on SGWT is performed by reconstruction of the decomposed data *y^N_θ_^* and 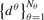: 
(15)
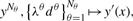

where *y*(*x*) represents input data, the superscript *θ* denotes the scale level, *d^θ^* is the wavelet coefficient at scale *θ*, *λ^θ^*is a parameter to determine the extent of smoothing at scale *θ*, *N_θ_* is the total number of scale and *y*′(*x*) represents the function of smoothed data. Small *λ* values at fine scales smooth the details of data, whereas large *λ* values at fine scales enhance the details. In order to automatically determine *λ* values in Equation (15), we use bivariate shrinkage (Sendur and Selesnick, 2002, 2003), which is the Bayesian estimation of the dependency between the neighboring wavelet coefficients.

In this study, we use *y*′ produced by the SGWT-based smoothing to obtain *S* and *E* according to Equations (13) and (14). This *S* is used in the Step 5 of Subsection 3.6. We also use *S* and *E* to make a list of the lengths of the regions and confine the model space *M* to this list.

## 4 RESULTS AND DISCUSSION

To demonstrate the effectiveness of the proposed method, we applied it to both synthetic and real genomic data and compared it with a previous approach.

### 4.1 Parameter setting

In the performance evaluations, we set the model and algorithmic parameters, as shown in Table 1, where *β*_0_ and *δ*_0_ are the initial *β* and *δ*, respectively. The model parameters define the differentially expressed regions we attempt to find with the proposed method; *γ*_min_ and *γ*_max_ define the region size, *z*_min_ and *z*_max_ define the extent of differential expression and *k* is the criterion of the neighborhood. The weighting factors *w* and *w_n_* define the balance of the effects of the data and prior energy terms; these parameters are determined experimentally so that the presented method detects the neighboring regions with the intervals appropriate for the users. The algorithmic parameters *β* and *δ* are determined according to the simulated annealing scheme; *β* and *δ* are, respectively, multiplied by 1.02 and 0.999 in each iteration. We set the parameter *v* from data according to 
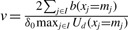
 in the first iteration. The parameters for the reduction of the region candidates described in Subsection 3.7 are also shown in Table 2. The proposed method takes ~1 s to analyze a set of 10 000-mer sequences by using a 2.0 GHz CPU computer.

**Table 1.** Model and algorithmic parameters for our differential analysis method

*γ*_min_	*γ*_max_	*z*_min_	*z*_max_	*k*
5	2000	4	40	25

*w*	*w_n_*	*β*_0_	*δ*_0_	

1	0.5	1	2

**Table 2.** Algorithmic parameters for the reduction process of the region candidates in Subsection 3.7

*t*	*N_θ_*
5	4

### 4.2 Performance evaluation on synthetic data

**Fig. 2.**
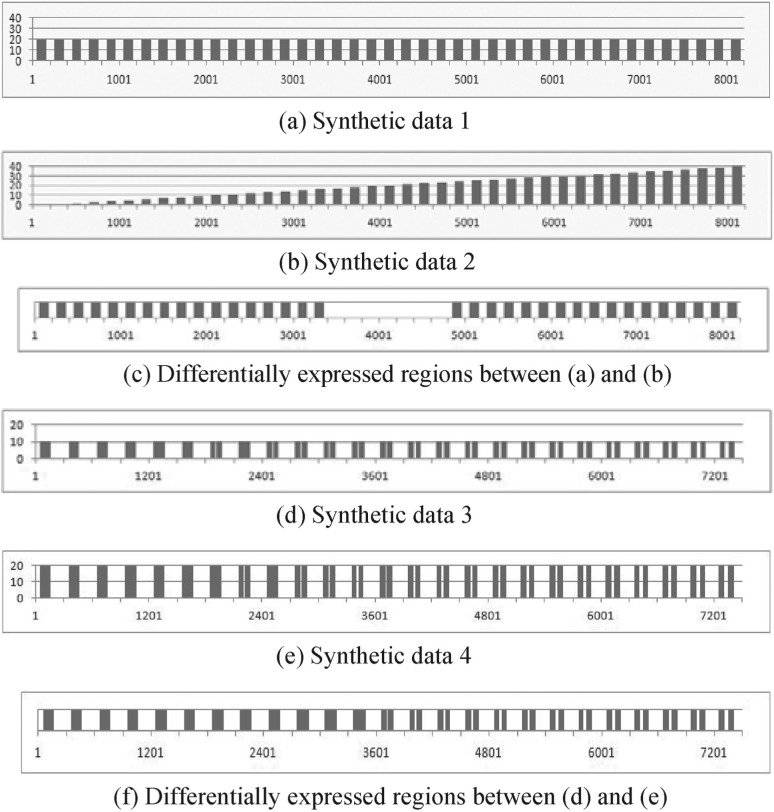
Applications of the proposed method to synthetic data. (**a**), (**b**), (**d**) and (**e**) show virtual expressions in a certain genomic region. Bars in (**c**) and (**f**) show differentially expressed regions. Heights of the bars in (**c**) and (**f**) do not have meaning

Figure 2 shows synthetic data and their analysis results by the presented method. Figure 2a, b, d and e denotes virtual expressions in a certain genomic region; the *x*-axis represents genomic position and the *y*-axis represents expression. Figure 2c demonstrates that it successfully detects differentially expressed regions and does not detect equally expressed regions between 2a and b. We can modulate the degree of difference it detects by setting the parameters *z*_min_ and *z*_max_ properly. Figure 2d, e and f illustrates the effect of the prior energy of our method. In Figure 2d and e, the distance between adjacent two regions becomes larger from left to right. If two differentially expressed regions are close to each other, our method detects one region; and if two differentially expressed regions are sufficiently away from each other, it detects two regions, as shown in Figure 2f. We can modulate the effect of the prior energy by setting the parameter *w* properly.

### 4.3 Differential analysis of TSS data

We also applied the proposed method to real genomic data. In this study, we used RNA expression of TSS of *Drosophila melanogaster* as real genomic data. In order to prepare base-level expression data, we aligned the 5′-SAGE reads, published at the database of TSS of *Drosophila* (Ahsan *et al.*, 2008), to *Drosophila* genome by using BLAT (Kent, 2002). We then counted the number of reads aligned at each nucleotide; we excluded the reads that were aligned to more than two locations in the genome or had more than four mismatches. We thus have RNAexpression of TSS at base resolution for each biological condition, including young female, young male, old female and old male.

**Fig. 3.**
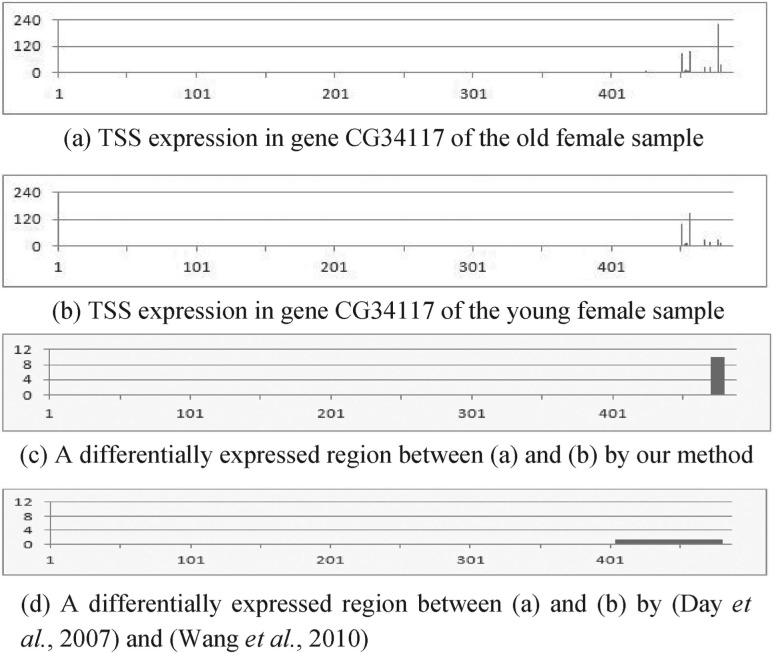
Application of the proposed method to real genomic data 1: RNA expression data of TSS in gene CG34117 of *Drosophila* of (**a**) the old female and (**b**) young female samples. (**c**) Differential analysis using the proposed method. It finds a differentially expressed (*z*-score *>* 4) region between (a) and (b). (**d**) Differential analysis using the previous methods. The *y*-axis represents *z*-score of each region in (c) and (d). *Z*-score between (a) and (b) in the whole gene is 1.2

**Fig. 4.**
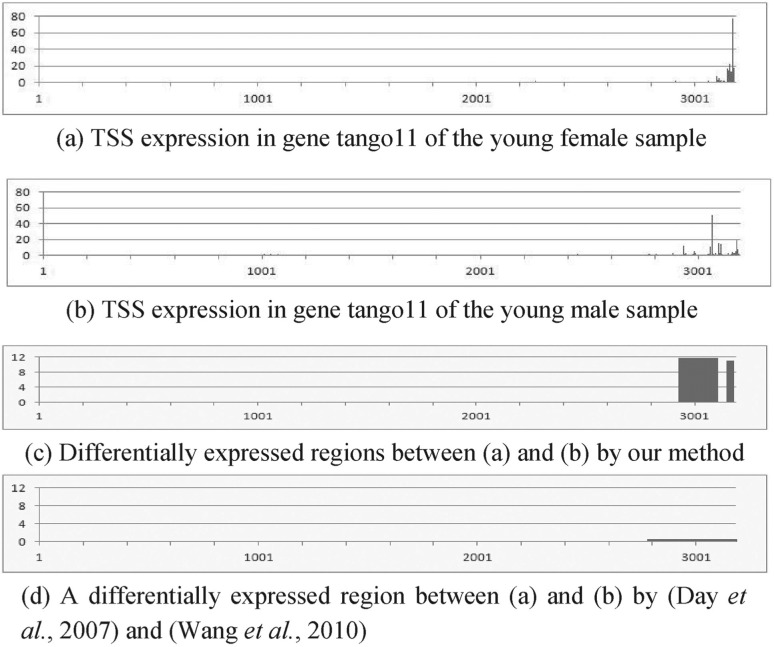
Application of the proposed method to real genomic data 2: RNA expression data of TSS in gene tango11 of *Drosophila* of (**a**) the young female and (**b**) young male samples. (**c**) Differential analysis using the proposed method. It finds differentially expressed (*z*-score *>* 4) regions between (a) and (b). (**d**) Differential analysis using the previous methods. The *y*-axis represents *z*-score of each region in (c) and (d). *Z*-score between (a) and (b) in the whole gene is 0.1

**Fig. 5.**
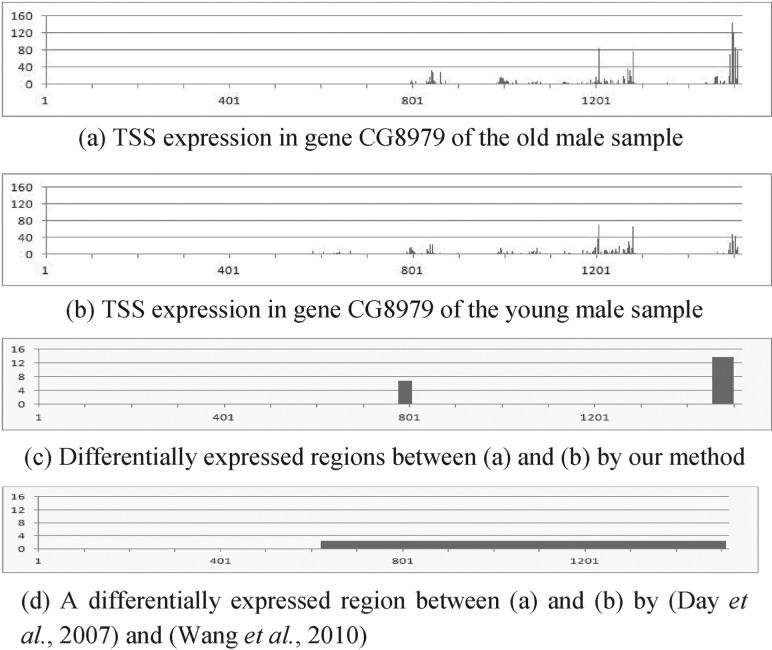
Application of the proposed method to real genomic data 3: RNA expression data of TSS in gene CG8979 of *Drosophila* of (**a**) the old male and (**b**) young male samples. (**c**) Differential analysis using the proposed method. It finds differentially expressed (*z*-score *>* 4) regions between (a) and (b). (**d**) Differential analysis using the previous methods. The *y*-axis represents *z*-score of each region in (c) and (d). *Z*-score between (a) and (b) in the whole gene is 1.9

**Fig. 6.**
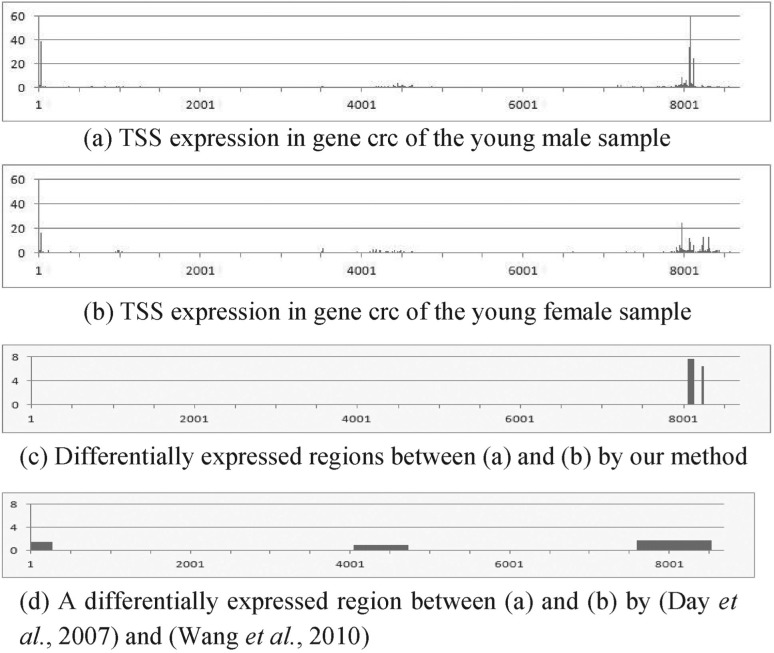
Application of the proposed method to real genomic data 4: RNA expression data of TSS in gene crc of *Drosophila* of (**a**) the young male and (**b**) young female samples. (**c**) Differential analysis using the proposed method. It finds differentially expressed (*z*-score *>* 4) regions between (a) and (b). (**d**) Differential analysis using the previous methods. The *y*-axis represents *z*-score of each region in (c) and (d). *Z*-score between (a) and (b) in the whole gene is 0.9

Figures 3–6 show four examples of RNA expression data of TSS and their differential analysis results. Figures X(a) and (b) (X = 3–6) represent the expression at each nucleotide, X(c) show differentially expressed regions detected by our method and their *z*-scores and X(d) denote segmentation results and their *z*-scores by a previous approach (Day *et al.*, 2007; Wang *et al.*, 2010). Although the four genes in these figures are not differentially expressed in the whole gene, they have differentially expressed smaller regions. This means that they have almost the same total expression in different biological conditions; however, they have distinct distributions of the expression. For example, Figure 3a has three large peaks; while 3b has two large peaks. This difference is biologically important because the peak shift in RNA expression is thought to be associated with the binding to DNA of different transcription factors due to environmental changes (Carninci *et al.*, 2006). The presented method can detect this difference; however, this difference is often not found by previous methods based on genome segmentation because they are indirect differential analysis methods. They usually depend on the degree of smoothing process and thus tend to fail to provide the regions of appropriate length in terms of differential analysis. For instance, because we set the window size of wavelet-based smoothing to 128 and the number of state of segmentation based on HMM to 2 to segment genes in this evaluation, we obtained a large region, as shown in Figure 3d, and thus failed to find the rightmost differentially expressed peak in 3a. In contrast, our method can locate the region containing that peak because it directly searches differentially expressed regions based on the difference of the expressions without genome segmentation and does not depend on the smoothing. The same applies to Figures 4–6. These examples demonstrate the effectiveness of our method.

In addition, although we can select smaller numbers for the window size of the smoothing to obtain smaller segments, we need to choose larger values for the state number of HMM in that case because the small window size produces many clusters with different expression levels. This means that we need a laborious process of trial and error for setting the state number to obtain a good segmentation; it is infeasible for genome analysis to select an appropriate state number in each gene. We thus have to use a large number for the window size so that we can set the state number to 2, which means regions of interest and regions of not interest. This is a serious drawback in the previous approach consisting of smoothing and HMM-based segmentation; in contrast, the proposed method is free from this problem because it does not depend on the genome segmentation irrelevant to differential expression analysis.

## 5 CONCLUSION

In this article, we have presented a novel method based on a marked point process model to find differentially expressed regions of arbitrary length in quantitative genomic data without depending on genome annotations. The proposed method is effective because it can locate differentially expressed regions that previous approaches, such as genome segmentation, fail to find. This is highly important for genome science because we currently have large amounts of quantitative genomic data at nucleotide resolution. For instance, we can study the relation between the type of promoters and RNA expression pattern in the regions near TSS to reveal the transcriptional regulation by using the presented method. In addition, it is important to note that the proposed method can be used to detect any type of regions of interest by incorporating suitable data energy and analyze all kinds of base-level quantitative genomic data.
